# “We regret to inform you that you did not match”: Reflections on how to improve the match experience

**DOI:** 10.36834/cmej.69322

**Published:** 2020-07-15

**Authors:** T.K. Fellows, S. Freiman, V. Ljubojevic, S. Saravanabavan

**Affiliations:** 1 Faculty of Medicine, University of Toronto, Ontario, Canada

## Abstract

There is increasing concern amongst stakeholders over the high numbers of unmatched Canadian Medical Graduates (CMGs), yet little is known from the perspective of those who go unmatched. We present an opinion-based narrative analysis examining the matching process by reflecting on the pre- and post-match period and provide suggestions related to the Canadian context from the unmatched perspective. The challenge in the pre-match period was a lack of transparency around elective availability, resident selection criteria, and what happens after going unmatched. For the post-matched period, we were challenged with decision-making during a time-sensitive period, scheduling post-match electives, handling our finances, and improving our future residency applications without feedback. We have tried to identify the most impactful issues we encountered as applicants and unmatched students, and offered suggestions to improve the applicant experience. In addition to sharing our reflection in going unmatched, we also highlight the positive side of this formative experience.

## Abstract (fr)

Les parties prenantes sont de plus en plus préoccupées par le nombre élevé de diplômés canadiens en médecine (DCM) qui ne sont pas jumelés. Or, l’on sait peu de choses sur leur perspective sur le sujet. Nous présentons une analyse narrative fondée sur les opinions qui examine le processus de jumelage en réfléchissant aux périodes précédant et suivant le jumelage. Nous fournissons également des suggestions pouvant s’appliquer aux individus non jumelés dans le contexte canadien. Le défirencontré en ce qui concerne la période précédant le jumelage tenait au manque de transparence en ce qui a trait à la disponibilité de stages à option, les critères de sélection des résidents et ce qui arrive aux étudiants une fois qu’ils ne sont pas jumelés. En ce qui concerne la période suivant le jumelage, nous avons eu de la difficulté à prendre des décisions étant donné la période de temps limitée ; planifier les stages à option après le jumelage ; gérer nos finances et améliorer notre processus d’application à un poste de résidence sans avoir obtenu de rétroaction. Nous avons tenté d’identifierles problèmes ayant eu le plus d’impact sur nous comme candidats et les étudiants non jumelés et nous avons proposé des suggestions afin d’améliorer l’expérience des candidats. En plus de partager nos réflexions sur le fait de ne pas être jumelé, nous mettons également en évidence le côté positif de cette expérience formative.

## Introduction

**Figure 1:**
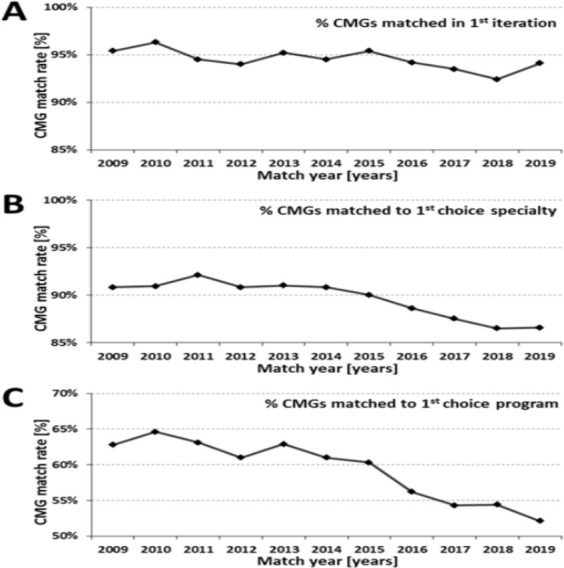
Residency match outcomes for Canadian medical graduates (CMGs) in the past 10 years. Of note, the y-axes have been adjusted to magnify and illustrate the change in matching to first choice specialty and program **A**. Overall CMG match rate in the first iteration of the Canadian Residency Matching Service (CaRMS). **B**. Percentage of CMGs matching to their first choice of specialty (at any school) in the first iteration of CaRMS. **C**. Percentage of CMGs matching to their first choice program (specialty and school) in the first iteration of CaRMS.

There seems to be a decline in Canadian Medical Graduate (CMG) residency match rates over the past 10 years, specifically matches to first choice specialties and programs (Figure 1). This has also been recognized as a concern by the Association of Faculties of Medicine of Canada (AFMC),^1^ who have critiqued the residency application process and provided recommendations for improvement. While there have been reflective accounts of going unmatched,^2^ we provide an opinion-based examination of the application process from the perspective of unmatched CMGs.

Using our experiences with the Canadian Residency Matching Service (CaRMS), this paper presents a narrative analysis of the matching process. We have chosen the most impactful issues we personally encountered during our applications and after we went unmatched, and offer suggestions that would have improved our experiences.

### Pre-match

We feel our experience could have been improved by increasing elective accessibility, improving transparency in the resident selection process, increasing diversity at the post-graduate medical education (PGME) selection committee, and implementing better expectation management around matching.

Elective experiences are essential and help determine our residency application success,^3^ yet arranging our electives was challenging. Two of us switched specialty focus a few months after elective applications opened, and consequently, struggled to secure relevant visiting electives. We found ourselves applying for electives that were already filled at the time of application. We encourage all schools to create an accessible document that would display real-time elective availability.

There is a lack of transparency in resident selection criteria. Students often make CaRMS application strategies based on informal advice and anecdotal evidence.^4^ For example, advice from residents to not “split electives” discouraged some of us from exploring parallel options. This year’s program descriptions provided more explicit selection criteria,^5^ which we feel has improved transparency. We would have appreciated information on how programs rank applicants, clarity around how somewhat peripheral factors like interview-adjacent social events and “red flags” during electives and interviews impact applicants’ rank.

We encourage ongoing efforts at the PGME level to ensure diverse selection committees. When there was representation of communities we identify with among the interviewers, we noticed it was easier to share our best qualities because we felt more accepted and secure. Increasing diversity in our medical community is a key step towards realizing equity in our education and patient health.

There is uncertainty around going unmatched. Rather than seeking information from their universities, many students reached out to us to learn about going unmatched. Programs need to better manage student expectations using early realistic conversations about not matching, creating awareness on the reality of healthcare human resource planning,^6^ and promoting the extra year as an opportunity for career exploration.

### Post-match

After going unmatched, we were challenged by making decisions during a time-sensitive period, scheduling electives, handling our finances, trying to improve our applications without feedback, and ultimately navigating the shame of going unmatched.

Once unmatched, we had to make important decisions, such as pursuing further electives through University of Toronto’s MD Extension Clerkship (MEC) program or graduating. Our university helped us navigate this time-sensitive period with in-person meetings with administration, providing an information package outlining our options, and facilitating easy access to counselling. Toronto’s MEC program allows their unmatched students to secure up to 32 weeks of elective time to expand their clinical experiences; other schools should adopt this or similar models.

Once in MEC, elective scheduling was complicated by navigating AFMC’s new MD Extension elective application category for unmatched students. In its first year, the rollout was vexed by delayed opening of this special applicant tab (i.e. unmatched in February, portal opened in May), non-functional portals for certain schools, and lost applications. Despite the glitches, this specialty application tab has been invaluable to us, and we hope future unmatched students will continue to have access.

Going unmatched is financially difficult. Deferring resident income, and paying for MEC tuition, electives and CaRMS fees extended our existing medical student debt. We encourage schools to advocate with provincial funding bodies for unmatched students to maintain their students’ status and continue having access to provincial loans.

In a profession requiring reflexivity as defined by the CanMEDS framework,^7^ we believe application feedback that would allow applicants to mature as medical professionals should be readily provided. None of us received feedback on our previous application, and consequently, we struggled with designing our fifth year to become stronger candidates in the next CaRMS cycle. We strongly encourage medical schools and PGME programs to provide focused feedback in future cycles.

During elective experiences, we all struggled with the shame of going unmatched. Conversations with matched colleagues were difficult, introductions to supervising staff during electives felt awkward, deferring convocation separated us from our cohort, and working as medical students with classmates who are now residents acted as a continuous reminder of our circumstances. This shame affected our wellness and institutional learning environment; we found benefit from access to mental health support, mentorship from previously unmatched students, and increased public engagement like the “Spots4Docs” campaign.^8^

## Conclusion

In this paper, we have aimed to highlight the most impactful issues we faced in the residency application process. Despite the challenges of going unmatched, we all took full advantage of our time in the MEC program. We had the chance to reflect on our future practice. While some of us changed which specialties we are applying to, some of us became even more confident in what we originally wanted to do. We have delved deeper into research and found new academic opportunities. We developed new skills, from certification in point-of-care ultrasound to learning a new dance style. We studied and travelled abroad. We felt liberated as the extended clerkship program allowed us to schedule experiences we enjoyed, allowed us to expand and strengthen our clinical skills and remain productive in preparation for future residency applications. We hope applications of future unmatched students will not be marked with a “red flag” but given a banner proclaiming resilience and perseverance.
